# Interrogating causal pathways linking genetic variants, small molecule metabolites, and circulating lipids

**DOI:** 10.1186/gm542

**Published:** 2014-03-28

**Authors:** So-Youn Shin, Ann-Kristin Petersen, Simone Wahl, Guangju Zhai, Werner Römisch-Margl, Kerrin S Small, Angela Döring, Bernet S Kato, Annette Peters, Elin Grundberg, Cornelia Prehn, Rui Wang-Sattler, H-Erich Wichmann, Martin Hrabé de Angelis, Thomas Illig, Jerzy Adamski, Panos Deloukas, Tim D Spector, Karsten Suhre, Christian Gieger, Nicole Soranzo

**Affiliations:** 1 Wellcome Trust Sanger Institute, Genome Campus, Hinxton CB10 1HH, UK; 2 MRC Integrative Epidemiology Unit (IEU), University of Bristol, Bristol, UK; 3 Institute of Genetic Epidemiology, Helmholtz Zentrum München, Neuherberg D-85764, Germany; 4 Research Unit of Molecular Epidemiology, Helmholtz Zentrum München, Neuherberg D-85764, Germany; 5 Institute of Epidemiology II, Helmholtz Zentrum München, Neuherberg D-85764, Germany; 6 German Center for Diabetes Research (DZD), Neuherberg, Germany; 7 Department of Twin Research & Genetic Epidemiology, King’s College London, London SE1 7EH, UK; 8 Discipline of Genetics, Faculty of Medicine, Memorial University of Newfoundland, Newfoundland, Canada; 9 Institute of Bioinformatics and Systems Biology, Helmholtz Zentrum München, Neuherberg D-85764, Germany; 10 Institute of Epidemiology I, Helmholtz Zentrum München, Neuherberg D-85764, Germany; 11 Institute of Epidemiology II, Helmholtz Zentrum München, Neuherberg D-85764, Germany; 12 Respiratory Epidemiology, Occupational Medicine and Public Health, Imperial College London, London SW3 6LR, UK; 13 Department of Human Genetics, McGill University, Montreal H3A 1A5, Canada; 14 Genome Quebec Innovation Centre, McGill University, Montreal H3A 1A5, Canada; 15 Institute of Experimental Genetics, Genome Analysis Center, Helmholtz Zentrum München, Neuherberg D-85764, Germany; 16 Institute of Medical Informatics, Biometry and Epidemiology, Chair of Epidemiology, Ludwig-Maximilians-Universität, München D-81377, Germany; 17 Klinikum Grosshadern, München D-81377, Germany; 18 Institute of Experimental Genetics, Life and Food Science Center Weihenstephan, Technische Universität München, Freising D-85354, Germany; 19 Hannover Unified Biobank, Hannover Medical School, Carl-Neuberg-Straße 1, 30625 Hannover, Germany; 20 Willian Harvey Research Institute, Barts and The London School of Medicine and Dentistry, Queen Mary University of London, London EC1M 6BQ, UK; 21 Princess Al-Jawhara Al-Brahim Centre of Excellence in Research of Hereditary Disorders (PACER-HD), King Abdulaziz University, Jeddah 21589, Saudi Arabia; 22 Department of Physiology and Biophysics, Weill Cornell Medical College in Qatar, Education City - Qatar Foundation, Doha, Qatar; 23 Department of Hematology, Long Road, Cambridge CB2 0PT, UK

## Abstract

**Background:**

Emerging technologies based on mass spectrometry or nuclear magnetic resonance enable the monitoring of hundreds of small metabolites from tissues or body fluids. Profiling of metabolites can help elucidate causal pathways linking established genetic variants to known disease risk factors such as blood lipid traits.

**Methods:**

We applied statistical methodology to dissect causal relationships between single nucleotide polymorphisms, metabolite concentrations, and serum lipid traits, focusing on 95 genetic loci reproducibly associated with the four main serum lipids (total-, low-density lipoprotein-, and high-density lipoprotein- cholesterol and triglycerides). The dataset used included 2,973 individuals from two independent population-based cohorts with data for 151 small molecule metabolites and four main serum lipids. Three statistical approaches, namely conditional analysis, Mendelian randomization, and structural equation modeling, were compared to investigate causal relationship at sets of a single nucleotide polymorphism, a metabolite, and a lipid trait associated with one another.

**Results:**

A subset of three lipid-associated loci (*FADS1*, *GCKR*, and *LPA*) have a statistically significant association with at least one main lipid and one metabolite concentration in our data, defining a total of 38 cross-associated sets of a single nucleotide polymorphism, a metabolite and a lipid trait. Structural equation modeling provided sufficient discrimination to indicate that the association of a single nucleotide polymorphism with a lipid trait was mediated through a metabolite at 15 of the 38 sets, and involving variants at the *FADS1* and *GCKR* loci.

**Conclusions:**

These data provide a framework for evaluating the causal role of components of the metabolome (or other intermediate factors) in mediating the association between established genetic variants and diseases or traits.

## Background

Recent technological advances allow for the collection of high-dimensional molecular phenotype datasets in thousands of individuals in a highly standardized manner. Metabolomics technologies based on mass spectrometry (MS) or nuclear magnetic resonance (NMR) enable the monitoring of hundreds of small molecule metabolites in tissues or body fluids [1-3]. Metabolites are intermediates in metabolic pathways, which can be used to obtain a snapshot of the physiological status of an individual at a given time point. These datasets are typically organized into metabolic correlation networks, which are mined to deduce unknown pathways from observed correlations, for instance to identify metabolic signatures of disease status [4].

An emerging application of quantitative or semi-quantitative technologies such as LC-MS-based metabolomics is their combination with genome-wide association data to discover genetic loci underlying variation in human metabolism. Genome-wide metabolomics scans based on hundreds of metabolite and lipid species measured using standardized high-throughput assays have to date identified over 100 independent loci for metabolites [5-14]. Importantly, several of the metabolite-associated loci correspond to loci previously associated with risk of disease or their risk factors such as Crohn’s disease, kidney disease, and serum lipids. These first studies have demonstrated the usefulness of large-scale metabolomics scans for formulating novel hypotheses on biochemical processes underpinning complex traits and diseases. Once correlations between a metabolite and a trait have been observed at a locus, however, the next challenge is to tease apart causal relations from shared environmental effects or confounding.

This study explored the application of statistical inference to dissect causal relationships at complex-trait loci where there is a concomitant association with one or more metabolites. The analysis was focused on: (1) a set of SNPs robustly associated with the four main circulating serum lipids in genome-wide association studies at the time of analysis, and including total cholesterol (TC), low-density lipoprotein cholesterol (LDL-C), high-density lipoprotein cholesterol (HDL-C), and triglycerides (TG) [15,16]; (2) 151 metabolites measured using the Biocrates platform [10]; and (3) the same four main serum lipids stated above. Briefly, subsets of the SNPs that have statistically significant associations with at least one metabolite and one lipid in our data were selected. Conditional analysis, Mendelian randomization (MR) [17], and structural equation modeling (SEM) [18-20] were then applied to the data to infer statistically causal relationships in each of SNP-metabolite-lipid sets previously defined.

The overarching aim of this study was to apply statistical approaches to interrogate causal relationships using genomic, metabolomic, and circulating lipid biomarker measures as an exemplar model. This provides a framework that can be applied in many other settings both in relation to metabolomics data as well as other -omic measures.

## Methods

### Study description

#### KORA

The Cooperative Health Research in the Region of Augsburg (KORA) study is a series of independent population-based epidemiological surveys and follow-up studies of participants living in the region of Augsburg, Southern Germany [21]. Blood samples for KORA F4 participants were collected between 2006 and 2008 in a standardized manner as previously described in detail [10].

##### Genotyping

For genotyping, 1,814 KORA F4 samples were randomly selected and genotyped using the Affymetrix Human SNP Array 6.0. After filtering out low call rate SNPs and SNPs violating Hardy-Weinberg Equilibrium (HWE), imputation was conducted using IMPUTE v0.4.2 [22] based on HapMap2.

##### Lipid measurement

Four serum lipid measurements (in mg/dl) were collected using the Dimension RxL (Dade Behring); total cholesterol was determined by cholesterol-esterase method (CHOL Flex, Dade-Behring, CHOD-PAP method), HDL-C cholesterol by the AHDL Flex (Dade-Behring, CHOD-PAP method after selective release of HDL-C), LDL-C cholesterol by the ALDL Flex (Dade Behring, CHOD-PAP method after colourless usage of all non-LDL-cholesterol) and triglycerides (TG) by the TGL Flex (Dade Behring, enzymatic colorimetric test, GPO-PAP method).

##### Metabolite measurement

A total of 3,044 KORA F4 samples were profiled using Biocrates Absolute*IDQ* Kit p150 across three periods of time (August/September 2008, November/December 2008, and March/April 2009; which were marked as three batches for the analysis). Finally, a total of 1,797 KORA F4 samples were available with genotypes, metabolite, and serum lipid measurements (Additional file 1: Table S1).

#### Twins UK

The TwinsUK cohort is an adult twin British registry recruited from the general population in the United Kingdom [23]. Blood samples collection has been described previously [9].

##### Genotyping

TwinsUK samples were genotyped using a combination of Illumina arrays (HumanHap300 [24,25], HumanHap610Q, 1 M-Duo and 1.2MDuo 1 M). For each dataset, the Illuminus calling algorithm [26] was used to assign genotypes (posterior probability ≥0.95) and applied the standardized data QC criteria based on: (1) call rate, heterozygosity, ethnicity, and relatedness (for sample exclusion); and (2) HWE, minor allele frequency, and call rate (for SNPs). After pair-wise concordance check and further visual inspection, the genotype datasets from different arrays were merged. Imputation was performed using the IMPUTE software package (v2) [22] using two reference panels, P0 (HapMap2, rel 22, combined CEU + YRI + ASN panels) and P1 (610 k+, including the combined HumanHap610 k and 1 M reduced to 610 k SNP content).

##### Lipid measurement

Serum lipids for TwinsUK samples were measured (in mmol/L) as described in [27] and the LDL-C values were derived from HDL-C and TG values using Friedewald’s equation. We converted all lipid measurements to mg/dl values to be consistent with KORA, by multiplying 38.67 for the LDL-C, HDL-C, and TC measurements and 87.5 for the TG measurement.

##### Metabolite measurement

Metabolite measurements were performed using the metabolomics platform Biocrates Absolute*IDQ* Kit p150 under an identical protocol as for the KORA study at the Genome Analysis Center of the Helmholtz Zentrum München. For 1,235 randomly selected TwinsUK samples with genotypes available, the metabolite measurements were conducted in two batches: one for 422 individuals in April 2009 and the other for 813 individuals in November 2009. One reference sample was included in each of the 10 plates run in the second batch, and metabolites were measured five times in each plate. These reference measurements were used for quality control purposes. After further QC (more details below), a total of 1,176 TwinsUK samples were available with metabolite, genotype, and serum-lipids measurements.

All the participants in both KORA and TwinsUK cohorts have provided informed consent and this study has been approved by Local Research Ethics Committee, Guy’s and St. Thomas’ Hospital Ethics Committee for TwinsUK, and Bayerische Landesärztekammer for KORA. Summary information for all the samples can be found in Additional file 1: Table S1.

### Metabolomics measurements and QC

#### Metabolite panel

The analyzed metabolite panel comprises 163 different metabolites, including 14 amino acids, hexoses (H1), free carnitine (C0), 40 acylcarnitines (Cx:y), hydroxylacylcarnitines (C(OH)x:y), and dicarboxylacylcarnitines (Cx:y-DC), 15 sphingomyelins (SMx:y) and N-hydroxylacyloylsphingosylphosphocholine (SM (OH)x:y), 77 phosphatidylcholines (PC, aa = diacyl, ae = acyl-alkyl), and 15 lyso-phosphatidylcholines. Quality parameters and quantification procedures were as described by us [28]. After quality control, 151 different metabolites remained in the dataset (Additional file 1: Table S2). Lipid side-chain composition is abbreviated as Cx:y, where x denotes the number of carbons in the side chain and y the number of double bonds. For example, ‘PC ae C32:1’ denotes an acyl-alkyl phosphatidylcholine with 32 carbons in the two fatty acid side chains and a single double bond in one of them. Full biochemical names are provided in Additional file 1: Table S1. The precise position of the double bonds and the distribution of the carbon atoms in different fatty acid side chains cannot be determined with this technology. In some cases, the mapping of metabolite names to individual masses can be ambiguous. For example, stereo-chemical differences are not always discernible, and neither are isobaric fragments. In such cases, possible alternative assignments are indicated.

#### Metabolite measurements in KORA and TwinsUK

Liquid handling of serum samples (10 μL) was performed with a Hamilton Star (Hamilton Bonaduz AG) robot, and samples were prepared for quantification using the Absolute*IDQ* Kit p150 (BIOCRATES Life Sciences AG). Sample analyses were done on 4000 Q TRAP LC/MS/MS System (AB Sciex) equipped with a Shimadzu Prominence LC20AD pump and a SIL-20 AC autosampler. The complete analytical process was performed using the MetIQ software package, which is an integral part of the Absolute*IDQ* kit. The MetIQ version 1.2.1r (Lithium), released in April 2010 was used, which incorporates an isotope correction. The experimental targeted metabolomics measurement technique is described in detail by US patent US 2007/0004044 [29] and in the manufacturer’s manuals. A summary of the method can be found in elsewhere [30-32], and a comprehensive overview of the field and the related technologies is given in [33]. Briefly, a targeted profiling scheme is used to quantitatively screen for known small-molecule metabolites using multiple reaction monitoring. Quantification of the metabolites of the biological sample is achieved by reference to appropriate internal standards. The method has been proven to conform to 21CFR (Code of Federal Regulations) Part 11, which implies proof of reproducibility within a given error range. It has been applied in different academic and industrial applications [11,33,34]. Concentrations of all analyzed metabolites are reported in μM.

#### Batch effects

The mean differences of the metabolomics measurements across different measurement batches were compared to assess the influence of possible batch effects due to calibration of the machines at periodical time points. To account for these differences in mean, a batch variable was included in all analyses of metabolomics data. For consistency this batch variable was applied to all metabolites independent of demonstration of significant batch effects.

#### Quality control

Quality control of the metabolomics datasets was conducted in two steps. In the first step the quality of all metabolites was controlled by their coefficient of variation (CV) and missing value rate. For CV calculation, one reference blood sample was measured five times on each plate across all 10 plates. The CV for each metabolite was calculated as follows:

$$\mathrm{CV}=\frac{\mathrm{sd}\;\;\left(\mathrm{all}\;\mathrm{five}\;\mathrm{reference}\;\mathrm{measurements}\right)}{\mathrm{mean}\;\left(\mathrm{all}\;\mathrm{five}\;\mathrm{reference}\;\mathrm{measurements}\right)}\;$$

The mean CV for each metabolite was computed from all 10 plates. All metabolites with a mean CV greater than 25% were excluded. In addition to this criterion, a maximal missing value rate of 5% was imposed. The second step of our quality control was removing outlying data points and outlying samples. This step was applied to log-transformed metabolites, which were consistently closer to normality than the untransformed metabolites based on the Anderson Darling test. Outlying data points were defined as values greater than 5 sd away from the mean for each metabolite. For each sample, two outlying data points were claimed to be independent if the correlation of corresponding metabolites was less than 70%. Samples with more than three independent outlying data points were excluded. For samples with less than, or equal to, three independent outlying data points, only the data points were excluded. Finally, all missing values were imputed using the R-package ‘mice’ [35], which applies a linear regression approach to estimate a distribution of each variable with missing values conditional on all the other variables in the same multivariate dataset, and replaces missing values with simulated values drawn from this distribution.

#### Data summary

A total of 163 metabolites were measured in 3,061 samples of KORA F4 and in 1,237 samples of TwinsUK. In the first step of quality control, 11 metabolites were excluded for having a CV higher than 25% and one metabolite for having more than 5% missing values (Additional file 1: Table S2). In the second step, 17 samples were discarded in KORA F4, due to their multiple independent outlying data points and two samples in TwinsUK. In addition, 419 and 254 outlying data points were treated as missing values in KORA F4 and TwinsUK, respectively. Together with the original missing data points, 0.09% of all data points were imputed in KORA F4 and 0.16% in the TwinsUK. After sample and metabolite exclusions, a total of 151 metabolites were available for analysis in 3,044 samples in KORA F4 and 1,235 samples in TwinsUK (among which 1,797 samples in KORA F4 and 1,176 in TwinsUK had available metabolite, genotype, and serum-lipids measurements).

### Candidate SNPs

The analysis focused on a total of 102 SNPs at 95 lipid-associated loci reported as primary association signals in a large-scale GWAS [16] for four lipid traits under the genome-wide significance threshold (*P* value ≤5 × 10^−8^) since our study would not have the same statistical power to detect additional novel lipid-associated loci with even smaller variances explained. Among the 102 SNPs, 52 were associated with TC, 37 with LDL-C, 47 with HDL-C, and 32 with TG in the original study. Many of these loci were associated with multiple lipid traits; for example, 41 were associated with two lipid traits, seven with three lipid traits, and six with all four lipid traits. Summary information for these SNPs measured in KORA and TwinsUK cohorts can be found in Additional file 1: Table S3.

### Statistical analyses

#### Metabolite and lipid trait transformation

The Anderson Darling test with and without log-transformation was used to test deviation from normality for metabolite values. The log-transformed metabolites were consistently closer to normality than the untransformed metabolites, and thus all metabolite measurements were log-transformed for analysis. The skewness of metabolites used in our causal analyses is reported in the Additional file 1: Table S8. Most metabolites had skewness between −0.5 and 0.5, indicating a symmetrical distribution, with the exception of PC aa C32:2 in KORA (skewness of −0.934) and five metabolites in TwinsUK. However, these small deviations from symmetry had no impact on the results and interpretation of causal relationships (data not shown), so no filtering or transformation were applied at this stage. For lipids, TG values were log-transformed to achieve normality. The distribution of LDL-C, HDL-C, and TC approximated normality and no transformation was applied.

#### Heritability

For each metabolite, the narrow sense heritability was estimated from 86 monozygotic and 245 dizygotic twin pairs in TwinsUK under the ACE model. The ACE model assumes that the phenotypic variance is influenced by additive genetic variation, common environmental effects, and unique environmental effects (or random effects), and infers the narrow sense heritability as the ratio of the estimated additive genetic variance to the phenotypic variance. The estimation was done by maximum likelihood methods implemented in OpenMx software [36].

#### Spearman’s correlation tests

Spearman’s correlation tests were used to identify correlated metabolite-lipid pairs, defined as *P* value <8.3 × 10^−5^ (Bonferroni corrected for 4 lipids and 151 metabolites) and the same direction of Spearman’s rho in both cohorts. We note that this correction over the number of tests may be over-conservative owing to highly correlated metabolite concentrations. Significant covariates (sex, age, and batch effect) were regressed out from metabolites and lipids prior to the correlation test. The computation of the *P* value and Spearman’s rho were done using the function ‘cor.test’ in R. Correlations were visualized by a heat map plot combined with a hierarchical clustering using the ‘heatmap.2’ function of the R-package ‘gplots’ [37] with default settings.

#### Single-trait association and meta-analysis

The association of the 102 candidate SNPs with all 151 metabolites was investigated under the linear model adjusting for age, batch, and sex, using SNPTEST and MERLIN (with -fastassoc option) in the KORA and TwinsUK sample, respectively. Summary statistics for the two cohorts were combined based on the inverse of the variance under the fixed effect meta-analysis model, and SNPs with *P* value <3.3 × 10^−6^ (=0.05 / (102 × 151)) in the meta-analysis and nominal association (*P* <0.05) in both cohorts were selected. Associations of the 102 candidate SNPs and main lipids were also tested using the same approach, and SNPs with *P* value <0.05 in the TwinsUK-KORA meta-analysis were retained for analysis.

#### SNP-MET-LIP sets

Each metabolite with its statistically significantly associated SNP and lipid trait (defined by the criteria above) was assigned to a unique SNP-MET-LIP dataset, where SNP denotes a genetic variant, MET denotes a metabolite, and LIP denotes a serum lipid trait. Only unrelated samples in TwinsUK (N = 845) were included for analysis. For metabolites and lipid traits, covariates adjustment were performed including age, sex, and batch effect using a linear regression model [16].

#### Conditional analysis

For each SNP-MET-LIP set, the association between SNP and LIP was tested under a linear regression model with and without adjustment for MET.

$$\begin{array}{l} \mathrm{Unadjusted}\;\mathrm{model}\;:y_{\mathrm{LIP}}=\alpha +\beta \cdot x_{\mathrm{SNP}}+\epsilon \\ \mathrm{Adjusted}\;\mathrm{model}\;\mathrm{for}\;\mathrm{the}\;\mathrm{metabolite}\;:y_{\mathrm{LIP}}=\alpha_{\mathrm{adj}}+\beta_{\mathrm{adj}}\cdot x_{\mathrm{SNP}}+\\ \;\gamma_{\mathrm{adj}}\cdot x_{\mathrm{MET}}+\epsilon_{\mathrm{adj}}\; \end{array}$$

To examine the influence of MET on SNP-LIP association, the *P* value between SNP and LIP in adjusted model was examined (in the way that *P* value ≥0.05 was considered as unlikely to have direct association) and the change of the estimated effect size of SNP was measured as follows.

$$\mathrm{Effect}\;\mathrm{size}\;\mathrm{change}\;:=\;\frac{{\hat{\beta }}_{\mathrm{adj}}-\hat{\beta }}{\hat{\beta }}\;$$

### Mendelian randomization

To estimate the causal effect of a metabolite on a lipid trait, Mendelian randomization (MR) [17,38] was applied to each SNP-MET-LIP set. Briefly in the MR approach, a genetic variant (G, here SNP) is used as an instrumental variable, which is not correlated with unknown confounders (U), to test a hypothesis that a variable (X, here MET) is causal to the outcome (Y, here LIP).

MR studies rest on three assumptions: (1) G is associated with X; (2) G is independent of U; and (3) G is independent of Y given X and U, that is, there is only one path from G to Y which is through X. For the estimation in MR, the Wald ratio, two-stage least squares and limited information maximum likelihood are commonly used, which are equivalent for a single instrument [39]. The Wald ratio method was applied here to estimate the unconfounded causal effect from MET to LIP [40] from the ratio of the regression coefficient of SNP in a linear regression of MET and LIP on SNP, respectively, under a simple linear model.

$${\hat{\beta }}_{\mathrm{MET}\to \mathrm{LIP}}=\frac{{\hat{\beta }}_{\mathrm{SNP}\to \mathrm{LIP}}}{{\hat{\beta }}_{\mathrm{SNP}\to \mathrm{MET}}}$$

The confidence interval of the unconfounded causal effect was computed using 1,000 bootstrap replicates [41] using the R-package ‘boot’.

### Structural equation modeling

**Figure 1:**
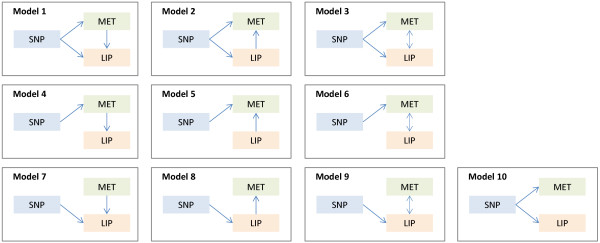
**SEM models.** The figure shows all 10 possible path models for a cross-associated set of a SNP, a metabolite or ratio, and a serum lipid, conditioned on the paths originating from the SNP [18]. Of these, only Models 4 to 10 were tested because Models 1 to 3 were overparameterized in our study (that is, they had zero degrees of freedom). Models 1 to 3 are also Markov equivalent and cannot be statistically distinguished.

SEM represents a generalization of the MR model. While MR tests the magnitude of an unconfounded effect under a given hypothesis on a causal relationship (for example, SNP → MET → LIP), SEM measures the likelihood of each of the possible hypotheses on path model implying a causal relationship, to select the best fitted path model. When a SNP and two traits are cross-associated with one another, 10 path models are suggested to be possible (Figure 1) [18]. Of these, only Models 4 to 10 were tested for SNP-MET-LIP sets because Models 1 to 3 in Figure 1 were overparameterized in our study (that is, they had zero degrees of freedom). Models 1 to 3 are also Markov equivalent and cannot be statistically distinguished as their maximized likelihood are the same [42-44]. It should be also noted that Model 4 in Figure 1 corresponds to the MR model, however, the estimation of Model 4 within the SEM framework would be done by the full information maximum likelihood method, rather than by the limited information maximum likelihood method that coincides with the MR we used above. The former maximizes the full joint likelihood and the latter the reduced likelihood only [39].

In details, the structural model can be denoted as

v=Av+u

where *v* is the vector of all the variables included in the model, *u* is the vector of residuals, and *A* is the matrix of the model coefficients. Under the same assumptions of a simple regression model (including independence, constant variance, and normality of the errors as well as linearity between dependent and independent variables), the expected covariance matrix Σ can be estimated as follows

$$\Sigma =E\left(vv^{T}\right)={\left(I-A\right)}^{-1}E\left(uu^{T}\right)\left(I-A\right).$$

The matrix Σ = Σ(θ) is a function of model parameter vector θ which includes model coefficients, measurement errors, and structural disturbances. Next, the observed covariance matrix *S* is computed directly from the variable values. Finally, the difference between expected and observed covariance matrices *Σ* and *S* is evaluated by Pearson’s chi-squared test (Goodness of Fit Test) under the null hypothesis that the model fits the observation. The test statistic is derived as

$$\ln\left|\Sigma \left(\theta \right)\right|+\mathrm{tr}\left(S\Sigma^{-1}\left(\theta \right)\right)-\ln\left|S\right|-p\sim X^{2}$$

where *p* is the number of variables included. All SEM analyses were performed by using the R-package ‘sem’ [45].

Once the fit of all possible path models was evaluated, the best fitted model was required to fit the following four criteria as defined previously [46-48]: (1) Goodness of Fit Test *P* value ≥0.05 (indicating how likely the hypothesis is, or how well the observed data fit the expectation of the model); (2) 0.9 < Goodness of Fit Index (GoFI) ≤1; (3) Root Mean Squared Error Approximation (RMSEA) ≤0.05; (4) smallest negative Bayesian Information Criterion (BIC). Where multiple models fit to the data, the best fitted model was selected if its BIC was at least two units smaller than the next lowest BIC [48], otherwise none was selected.

### Software programs

Most analyses were carried out using publically available packages in the R environment. SNP-metabolite association analyses were carried out using SNPTEST and MERLIN. Heritability estimation was carried out in OpenMx.

## Results

**Figure 2:**
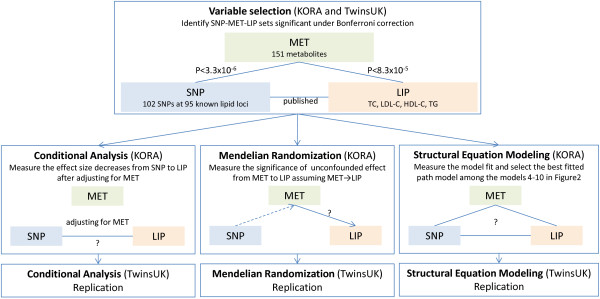
Study design.

The study design is shown in Figure 2. The Biocrates metabolomics profiling described in Illig *et al.*[10] was extended to an additional 813 TwinsUK samples. After stringent quality controls, a complete set of data for 151 metabolite concentrations (Additional file 1: Table S2) and four main serum lipid traits (TC, LDL-C, HDL-C, and TG) collected at the same time point became available for 1,797 and 1,176 individuals from the KORA (Germany) and TwinsUK (UK) samples, respectively (Additional file 1: Table S1).

To quantify the genetic basis of each metabolite concentration, the proportion of the heritable variance was estimated from 86 monozygotic and 245 dizygotic twin pairs in TwinsUK samples under the ACE model. A total of 96 metabolites were observed to be moderately to highly heritable (68 with 25% ≤ h^2^ < 50% and 28 with h^2^ ≥ 50%) (Additional file 1: Table S2) confirming a broad genetic basis for small metabolites.

### Metabolite levels are associated with four main serum lipids

The Biocrates metabolite panel is particularly informative for the study of lipid metabolism as it assays predominantly lipid species including sphingolipids and glycerophospholipids, besides amino acids. Correlation between metabolites and the four main serum lipid traits were assessed using Spearman’s correlation test, showing that all 151 metabolites were associated with at least one of the four lipid traits, and 30 metabolites with all lipid traits, at a stringent significance cutoff (*P* value <8.3 × 10^−5^; Additional file 1: Table S4). In particular, 94 metabolites were statistically significantly associated with TC, 84 with LDL-C, 71 with HDL-C, and 55 with TG in both KORA and TwinsUK samples. A heat map plot of metabolite-lipid correlation combined with a hierarchical clustering highlights six main groups of metabolites showing similar patterns of correlation (Additional file 2: Figure S1).

### Metabolite levels are associated with known lipid SNPs

Genetic associations between 151 metabolites and 102 SNPs at 95 known lipid loci [16] were further tested. Three loci, namely *FADS1*, *GCKR*, and *LPA*, were associated with at least one metabolite in the combined KORA and TwinsUK dataset (*P* value <3.3 × 10^−6^, Table 1). SNP rs174546 in *FADS1* was statistically significantly associated with concentrations of 34 different phosphatidylcholines (among which the strongest association was observed at PC aa C38:4 with Beta = −0.138 (SE = 0.007) and *P* value = 6.22 × 10^−83^), rs1260326 in *GCKR* was associated with the phosphatidylcholine PC aa C40:5 (Beta = 0.037 (0.008) and *P* value = 1.26 × 10^−6^) and rs1564348 in *LPA* with carnitines C3 (Beta = 0.053 (0.011) and *P* value = 4.94 × 10^−7^) and C8:1 (Beta = 0.09 (0.017) and *P* value = 6.28 × 10^−8^). Among them, the phosphatidylcholine PC aa C40:5 was associated with both rs174546 in *FADS1* and rs1260326 in *GCKR.*

**Table 1:** Association summary statistics

**Locus & SNP (effect/other allele)**	**Metabolite**	**Meta-analysis**			**KORA**			**TwinsUK**		
		**Beta**	**(SE)**	***P*** **value**	**Beta**	**(SE)**	***P*** **value**	**Beta**	**(SE)**	***P*** **value**
***GCKR*** **rs1260326 (T/C)**	PC aa C40:5	0.037	(0.008)	1.26 × 10^−6^	0.032	(0.009)	3.19 × 10^−4^	0.047	(0.014)	8.09 × 10^−4^
***LPA*** **rs1564348 (T/C)**	C3	0.053	(0.011)	4.94 × 10^−7^	0.049	(0.013)	1.15 × 10^−4^	0.062	(0.019)	1.24 × 10^−3^
	C8:1	0.09	(0.017)	6.28 × 10^−8^	0.064	(0.02)	1.60 × 10^−3^	0.143	(0.029)	4.86 × 10^−7^
***FADS1 *** **rs174546 (T/C)**	PC aa C32:0	−0.038	(0.006)	3.69 × 10^−10^	−0.039	(0.007)	4.21 × 10^−8^	−0.036	(0.012)	1.70 × 10^−3^
	PC aa C32:2	0.072	(0.012)	5.15 × 10^−9^	0.091	(0.017)	9.24 × 10^−8^	0.051	(0.018)	5.60 × 10^−3^
	PC aa C34:2	0.038	(0.005)	2.03 × 10^−13^	0.037	(0.006)	2.13 × 10^−10^	0.044	(0.012)	1.82 × 10^−4^
	PC aa C34:3	0.041	(0.008)	8.24 × 10^−7^	0.04	(0.01)	5.15 × 10^−5^	0.042	(0.015)	5.08 × 10^−3^
	PC aa C34:4	−0.100	(0.01)	1.45 × 10^−23^	−0.106	(0.012)	3.24 × 10^−17^	−0.09	(0.017)	7.61 × 10^−8^
	PC aa C36:2	0.043	(0.006)	6.32 × 10^−14^	0.045	(0.006)	3.75 × 10^−12^	0.034	(0.012)	4.01 × 10^−3^
	PC aa C36:3	0.055	(0.006)	5.48 × 10^−19^	0.053	(0.007)	1.13 × 10^−13^	0.058	(0.012)	3.38 × 10^−6^
	PC aa C36:4	−0.113	(0.006)	6.36 × 10^−69^	−0.112	(0.007)	1.21 × 10^−48^	−0.116	(0.013)	1.49 × 10^−19^
	PC aa C36:5	−0.129	(0.012)	8.72 × 10^−26^	−0.143	(0.016)	8.29 × 10^−20^	−0.105	(0.02)	1.12 × 10^−7^
	PC aa C36:6	−0.054	(0.011)	1.52 × 10^−6^	−0.051	(0.014)	2.34 × 10^−4^	−0.059	(0.019)	1.82 × 10^−3^
	PC aa C38:4	−0.138	(0.007)	6.22 × 10^−83^	−0.136	(0.008)	5.47 × 10^−56^	−0.144	(0.014)	2.14 × 10^−26^
	PC aa C38:5	−0.106	(0.007)	4.79 × 10^−51^	−0.108	(0.008)	4.48 × 10^−36^	−0.102	(0.013)	2.14 × 10^−14^
	PC aa C40:4	−0.075	(0.008)	9.54 × 10^−20^	−0.075	(0.01)	6.79 × 10^−14^	−0.076	(0.015)	2.14 × 10^−7^
	PC aa C40:5	−0.075	(0.008)	2.29 × 10^−21^	−0.075	(0.01)	8.02 × 10^−15^	−0.075	(0.014)	1.18 × 10^−7^
	PC aa C40:6	−0.050	(0.009)	1.21 × 10^−7^	−0.045	(0.012)	9.55 × 10^−5^	−0.058	(0.016)	2.74 × 10^−4^
	PC aa C42:0	−0.042	(0.009)	1.14 × 10^−6^	−0.035	(0.01)	7.54 × 10^−4^	−0.055	(0.015)	2.13 × 10^−4^
	PC aa C42:1	−0.065	(0.008)	6.13 × 10^−15^	−0.062	(0.01)	4.31 × 10^−10^	−0.075	(0.016)	2.12 × 10^−6^
	PC aa C42:4	−0.065	(0.007)	1.82 × 10^−20^	−0.064	(0.007)	1.15 × 10^−17^	−0.067	(0.02)	6.77 × 10^−4^
	PC aa C42:6	−0.050	(0.007)	3.05 × 10^−14^	−0.05	(0.008)	2.70 × 10^−10^	−0.05	(0.012)	4.05 × 10^−5^
	PC ae C36:2	0.060	(0.008)	1.58 × 10^−15^	0.07	(0.009)	5.04 × 10^−15^	0.034	(0.014)	1.41 × 10^−2^
	PC ae C36:3	0.069	(0.007)	1.11 × 10^−22^	0.076	(0.008)	1.87 × 10^−19^	0.051	(0.013)	8.28 × 10^−5^
	PC ae C36:4	−0.066	(0.007)	9.79 × 10^−20^	−0.058	(0.009)	2.46 × 10^−11^	−0.082	(0.013)	1.08 × 10^−10^
	PC ae C36:5	−0.096	(0.008)	1.22 × 10^−37^	−0.088	(0.009)	1.09 × 10^−22^	−0.116	(0.014)	3.38 × 10^−17^
	PC ae C38:4	−0.081	(0.006)	8.79 × 10^−40^	−0.076	(0.007)	5.96 × 10^−26^	−0.094	(0.012)	2.11 × 10^−14^
	PC ae C38:5	−0.076	(0.006)	1.72 × 10^−34^	−0.071	(0.007)	1.30 × 10^−21^	−0.092	(0.012)	5.76 × 10^−15^
	PC ae C38:6	−0.047	(0.007)	1.95 × 10^−10^	−0.041	(0.009)	2.91 × 10^−6^	−0.063	(0.014)	3.93 × 10^−6^
	PC ae C40:1	−0.062	(0.008)	8.54 × 10^−17^	−0.067	(0.009)	1.85 × 10^−14^	−0.049	(0.015)	9.82 × 10^−4^
	PC ae C40:4	−0.066	(0.006)	1.60 × 10^−25^	−0.064	(0.007)	3.23 × 10^−20^	−0.076	(0.016)	1.38 × 10^−6^
	PC ae C40:5	−0.065	(0.006)	2.60 × 10^−26^	−0.063	(0.007)	1.38 × 10^−19^	−0.076	(0.014)	4.91 × 10^−8^
	PC ae C40:6	−0.036	(0.008)	2.14 × 10^−6^	−0.029	(0.009)	9.86 × 10^−4^	−0.051	(0.014)	1.67 × 10^−4^
	PC ae C42:1	−0.048	(0.008)	2.12 × 10^−9^	−0.047	(0.009)	7.56 × 10^−8^	−0.052	(0.02)	8.09 × 10^−3^
	PC ae C42:5	−0.062	(0.006)	1.74 × 10^−22^	−0.057	(0.008)	4.97 × 10^−14^	−0.075	(0.012)	1.59 × 10^−9^
	PC ae C44:5	−0.071	(0.008)	1.08 × 10^−19^	−0.066	(0.009)	2.49 × 10^−12^	−0.081	(0.014)	6.64 × 10^−9^
	PC ae C44:6	−0.079	(0.008)	6.01 × 10^−23^	−0.075	(0.01)	3.47 × 10^−14^	−0.088	(0.014)	1.58 × 10^−10^

Summary statistics for the three loci selected for having a metabolite statistically significantly associated with a SNP and at least one lipid.

### Metabolites mediate some lipid pathways

Based on the association result, all 38 significant SNP-MET-LIP sets were selected (that is, where a metabolite was statistically significantly associated with a SNP and a lipid; Table 2). For each SNP-MET-LIP set, three different statistical approaches were used to test the hypothesis that MET might mediate SNP → LIP pathway.

**Table 2:** Results of conditional analysis, Mendelian randomization and structural equational modeling for the 38 significant SNP-MET-LIP sets

		**Conditional analysis**						**Mendelian randomization**		**Structural equation modeling**	
		**KORA**			**TwinsUK**			**KORA**	**TwinsUK**	**KORA**	**TwinsUK**
**Locus**	**SNP - MET - LIP**	**Beta**	**Beta**	**Beta**	**Beta**	**Beta**	**Beta**	**95% CI for Beta (MET → LIP using SNP as an IV)**	**90% CI for Beta (MET → LIP using SNP as an IV)**	**Best fitted model**	**Best fitted model**
		**(LIP ~ SNP)**	**(LIP ~ SNP + MET)**	**Changes**	**(LIP ~ SNP)**	**(LIP ~ SNP + MET)**	**Changes**				
***GCKR***	rs1260326 - PC aa C40:5 - TC	2.789	0.685	**−75%**	4.770	2.747	**−42%**	**0.34, 177.04**	−21.33, 168.93	Model 4 (SNP → MET → LIP)	Model 4 (SNP → MET → LIP)
	rs1260326 - PC aa C40:5 - TG	0.081	0.054	**−33%**	0.075	0.058	**−24%**	**0.28, 3.86**	−0.38, 2.30	Model 8 (SNP → LIP → MET)	
***LPA***	rs1564348 - C3 - HDL-C	1.095	1.640	50%	0.248	1.171	372%	−26.39, 46.60	−50.74, 35.81		
	rs1564348 - C8:1 - HDL-C	1.095	1.328	21%	0.248	1.319	432%	−24.00, 35.48	−16.69, 16.23		
***FADS1***	rs174546 - PC aa C32:0 - TG	0.043	0.061	43%	0.048	0.050	3%	−2.02, 0.35	−2.45, 2.68		Model 10 (MET ← SNP → LIP)
	rs174546 - PC aa C32:2 - TG	0.043	0.017	**−61%**	0.048	0.034	**−29%**	**0.02, 0.88**	−0.59, 1.56	Model 4 (SNP → MET → LIP)	Model 4 (SNP → MET → LIP)
	rs174546 - PC aa C34:2 - TG	0.043	0.006	**−87%**	0.048	0.037	**−22%**	**0.20, 2.25**	−0.52, 1.75	Model 4 (SNP → MET → LIP)	Model 4 (SNP → MET → LIP)
	rs174546 - PC aa C34:3 - TG	0.043	0.021	**−50%**	0.048	0.035	**−27%**	−0.41, 2.08	−1.09, 1.87	Model 4 (SNP → MET → LIP)	Model 4 (SNP → MET → LIP)
	rs174546 - PC aa C34:4 - TG	0.043	0.102	139%	0.048	0.076	58%	−0.77, 0.04	−1.13, 0.44		
	rs174546 - PC aa C36:2 - TG	0.043	0.004	**−90%**	0.048	0.041	**−15%**	**0.14, 1.78**	−1.20, 2.20	Model 4 (SNP → MET → LIP)	Model 4 (SNP → MET → LIP)
	rs174546 - PC aa C36:3 - TG	0.043	−0.016	**−138%**	0.048	0.022	**−54%**	**0.20, 1.55**	−0.10, 1.43	Model 4 (SNP → MET → LIP)	Model 4 (SNP → MET → LIP)
	rs174546 - PC aa C36:4 - TG	0.043	0.157	268%	0.048	0.084	75%	**−0.72, −0.02**	−0.83, 0.07		
	rs174546 - PC aa C36:5 - TG	0.043	0.077	79%	0.048	0.054	13%	−0.54, 0.03	−0.95, 0.12		Model 10 (MET ← SNP → LIP)
	rs174546 - PC aa C36:6 - TG	0.043	0.060	40%	0.048	0.053	10%	−1.52, 0.73	−1.92, 2.04		
	rs174546 - PC aa C38:4 - TG	0.043	0.177	315%	0.048	0.097	102%	−0.59, 0.01	−0.67, 0.06		
	rs174546 - PC aa C38:5 - TG	0.043	0.131	206%	0.048	0.069	44%	**−0.74, −0.02**	−0.96, 0.07		
	rs174546 - PC aa C40:4 - TG	0.043	0.103	140%	0.048	0.076	58%	−1.10, 0.13	−1.27, 0.44		
	rs174546 - PC aa C40:5 - TG	0.043	0.115	168%	0.048	0.077	60%	−1.07, 0.15	−1.22, 0.33		
	rs174546 - PC aa C40:6 - TG	0.043	0.071	65%	0.048	0.055	14%	−1.84, 0.84	−2.20, 2.10		
	rs174546 - PC aa C42:0 - TG	0.043	0.025	**−41%**	0.048	0.030	**−37%**	−2.62, 0.72	−1.73, 0.53	Model 4 (SNP → MET → LIP)	Model 4 (SNP → MET → LIP)
	rs174546 - PC aa C42:1 - TG	0.043	0.016	**−62%**	0.048	0.030	**−38%**	**−1.39, −0.07**	−1.24, 0.15	Model 4 (SNP → MET → LIP)	Model 4 (SNP → MET → LIP)
	rs174546 - PC aa C42:4 - TG	0.043	0.054	26%	0.048	0.051	6%	−1.29, 0.03	−1.20, 0.63		Model 10 (MET ← SNP → LIP)
	rs174546 - PC aa C42:6 - TG	0.043	0.067	57%	0.048	0.048	**0%**	−1.77, 0.33	−1.76, 0.48		Model 10 (MET ← SNP → LIP)
	rs174546 - PC ae C36:2 - TG	0.043	0.058	36%	0.048	0.054	12%	−0.06, 1.14	−3.89, 2.77		
	rs174546 - PC ae C36:3 - TG	0.043	0.069	61%	0.048	0.062	29%	−0.01, 1.03	−0.76, 1.89		
	rs174546 - PC ae C36:4 - TG	0.043	0.044	2%	0.048	0.037	**−23%**	−1.39, 0.11	−1.13, 0.10		Model 4 (SNP → MET → LIP)
	rs174546 - PC ae C36:5 - TG	0.043	0.031	**−29%**	0.048	0.016	**−66%**	**−0.93, −0.07**	−0.84, 0.02	Model 4 (SNP → MET → LIP)	Model 4 (SNP → MET → LIP)
	rs174546 - PC ae C38:4 - TG	0.043	0.036	**−16%**	0.048	0.044	**−8%**	**−1.10, −0.06**	−1.01, 0.07		Model 10 (MET ← SNP → LIP)
	rs174546 - PC ae C38:5 - TG	0.043	0.030	**−30%**	0.048	0.022	**−55%**	**−1.18, −0.05**	−1.02, −0.01	Model 4 (SNP → MET → LIP)	Model 4 (SNP → MET → LIP)
	rs174546 - PC ae C38:6 - TG	0.043	0.039	**−10%**	0.048	0.030	**−37%**	−1.99, 0.18	−1.52, 0.13		Model 4 (SNP → MET → LIP)
	rs174546 - PC ae C40:1 - TG	0.043	0.056	30%	0.048	0.046	**−3%**	−1.19, 0.10	−2.02, 1.04		Model 10 (MET ← SNP → LIP)
	rs174546 - PC ae C40:4 - TG	0.043	0.013	**−71%**	0.048	0.049	2%	**−1.36, −0.08**	−1.20, 0.23	Model 4 (SNP → MET → LIP)	Model 10 (MET ← SNP → LIP)
	rs174546 - PC ae C40:5 - TG	0.043	0.014	**−68%**	0.048	0.042	**−13%**	**−1.35, −0.05**	−1.26, 0.19	Model 4 (SNP → MET → LIP)	Model 4 (SNP → MET → LIP)
	rs174546 - PC ae C40:6 - TG	0.043	0.037	**−13%**	0.048	0.034	**−29%**	−2.90, 2.54	−1.99, 0.64	Model 4 (SNP → MET → LIP)	Model 4 (SNP → MET → LIP)
	rs174546 - PC ae C42:1 - TG	0.043	0.060	41%	0.048	0.051	6%	−1.81, 0.26	−1.74, 1.72		Model 10 (MET ← SNP → LIP)
	rs174546 - PC ae C42:5 - TG	0.043	−0.003	**−106%**	0.048	0.031	**−36%**	**−1.43, −0.11**	−1.35, 0.15	Model 4 (SNP → MET → LIP)	Model 4 (SNP → MET → LIP)
	rs174546 - PC ae C44:5 – TG	0.043	−0.001	**−102%**	0.048	0.022	**−54%**	**−1.26, −0.14**	−1.16, 0.12	Model 4 (SNP → MET → LIP)	Model 4 (SNP → MET → LIP)
	rs174546 - PC ae C44:6 – TG	0.043	−0.007	**−117%**	0.048	0.006	**−87%**	**−1.10, −0.10**	−1.02, −0.05	Model 4 (SNP → MET → LIP)	Model 4 (SNP → MET → LIP)

Effect size declines in conditional analysis, confidence intervals not containing 0 in Mendelian randomization, and Model 4 reported as the best fitted model in structural equation modeling in each cohort were highlighted in bold to provide evidence for the mediating role of MET in SNP-LIP pathways. SEM Model 4: SNP → MET → LIP; Model 8: SNP → LIP → MET; Model 10: SNP → MET; SNP → LIP.

First, the SNP-LIP association was conditioned on MET under a linear regression model in each SNP-MET-LIP set. A total of 19 metabolites associated with loci *GCRK* and *FADS1* resulted in marked declines of effect sizes in the metabolite-adjusted model (Table 2 and Additional file 1: Table S5). For example, the association between rs1260326 in *GCKR* and TC showed a 66% decrease in the effect size (from 3.274 mg/dl per copy of allele T, *P* value = 0.00429 to 1.125 mg/dl, *P* value = 0.275) after adjusting for PC aa C40:5. These observations were compatible with the hypothesis that these metabolites may mediate the lipid pathways.

**Figure 3:**
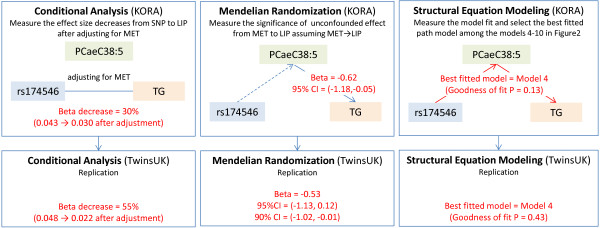
**Three different statistical analyses to test the hypothesis that a metabolite mediates the *FADS1*** **→ TG pathway.** The rs174546-T allele in *FADS1* locus is associated with both triglycerides and a small molecule metabolite, PC ae C38:5. We have tested the hypothesis that a metabolite mediates the lipid pathway using three different statistical approaches. The conditional analysis (left) confirmed that the effect size of rs174546 on triglyceride decreased conditional on PC ae C38:5 in both KORA and TwinsUK cohorts (top and bottom). The MR (middle) estimated a statistically significant causal effect of PC ae C38:5 on triglyceride, which however was not replicated in TwinsUK at 5% significance level, perhaps due to the small sample size (KORA = 1,797 and unrelated TwinsUK = 845). The SEM (right) showed that out of all possible models tested, the model 4 (rs174546 → PC ae C38:5 → trycliceride) was the best fitted one in both cohorts.

As a second approach, MR analysis was used to estimate the unconfounded causal effect of a metabolite on a lipid. For each SNP-MET-LIP set, the causal effect was estimated by the Wald method and its confidence interval was generated based on 1,000 bootstrap replicates. In KORA, 17 SNP-MET-LIP sets showed a causal relationship between MET and LIP (that is, MET → LIP) at the 5% significance level, however, none of them were replicated in TwinsUK at the same level of significance (although two of them were significant at 10% significance level and in need of further analysis in a larger dataset) (Table 2 and Additional file 1: Table S6). For example, by using rs174546 in *FADS1* as an instrumental variable, the unconfounded causal effect of PC ae C38:5 onto TG was estimated to be −0.62 (95% CI = (−1.18, −0.05)) in KORA, but only −0.53 (90% CI = (−1.02, −0.01)) in a set of unrelated TwinsUK individuals (Figure 3).

Lastly, SEM was applied to test a broader range of possible paths in each SNP-MET-LIP set. In a total of 15 SNP-MET-LIP sets, the best fitted model was shown to be Model 4 (which corresponds to the path tested by MR) assuming SNP → MET → LIP (Figure 1) in both KORA and TwinsUK. For example, in a set composed of rs174546 in *FADS1*, PC ae C38:5 and TG, only Model 4 showed Goodness of Fit Test *P* value ≥0.05 in both cohorts (Figure 3). This set also satisfied other criteria to be selected as the best fitted model; such as showing 0.9 < GoFI ≤1, RMSEA ≤0.05 and smallest negative BIC (Additional file 1: Table S7). Thus the SEM analysis supports the model tested by MR that phosphatidylcholines may mediate associations of *GCKR* to TC and *FADS* to TG (Table 2 and Additional file 1: Table S7).

## Discussion

Blood lipid levels are major risk factors for coronary artery disease (CAD) and myocardial infarction (MI), and targets for therapeutic intervention. Recent large scale meta-analyses of genome-wide association scans (GWAS) totaling >100,000 individuals has identified a total of 95 independent and common loci statistically significantly associated with at least one of the four main lipid traits (TC, LDL-C, HDL-C, and TG) [15,16]. Some of these loci are mapped to genes that are well known therapeutic targets [49-51], but for the majority, little is known in terms of their biological function or their value as therapeutic targets. Further characterization of the pathways via which these loci may influence lipid species is a necessary step towards evaluating their therapeutic potential.

In this study, the potential roles of metabolites as intermediate phenotypes of the four main lipid traits were examined. First, we showed that all 151 small metabolites profiled on the Biocrates metabolite panel were statistically significantly associated with lipid traits in two independent cohorts. Second, we demonstrated that 37 of these metabolites were robustly associated with variants at three different lipid-associated loci, including one metabolite associated with two loci, highlighting both known and potential new biochemical correlates (summarized in Table 3). Third, we applied a statistical framework composed of conditional analysis, MR, and SEM to investigate the role of metabolites in lipid pathways, and showed that one or more metabolites potentially mediate the SNP-lipid association at two loci, *FADS1* and *GCKR* (both statistically significant by SEM, and *FADS1* suggestively by MR).

**Table 3:** Summary of known evidence or hypothesis on the functional and biological role of metabolites for each of the three lipid loci

**Locus**	**Metabolite class**	**Functional and biological evidence**
***GCKR***	Phosphatidylcholine	*GCKR* encodes a glucokinase regulatory protein that inhibits glucokinase in liver and pancreatic islet cells by binding non-covalently to form an inactive complex with the enzyme. The locus has been shown to have a pleiotropic effect on multiple cardio-metabolic phenotypes [15,24,52-56]. We postulate here that GCKR SNPs affect TC through regulation of phosphatidylcholine metabolism, a hypothesis that needs to be validated in experimental settings.
***LPA***	Carnitine	A connection between Lp(a) and carnitine has been shown before. Derosa *et al.*[57] observed a statistically significantly decreased plasma Lp(a) concentration after L-carnitine intake of up to six month . Moreover, after a coadministration of simvastatin and carnitine the reduction in Lp(a) was significanty greater than after simvastatin medication alone [58].
***FADS1***	Phosphatidylcholine	The *FADS1-2-3* gene cluster encodes for fatty acid desaturase enzymes regulating the desaturation of fatty acids by adding double bonds between carbons of the fatty acyl chain [59-61]. Whereas FADS1 modifies the efficiency of the fatty acid delta-5 desaturase reaction, FADS2 modifies the fatty acid delta-6 desaturase reaction. GWAS of polyunsaturated fatty acids have shown associations between different fatty acids and the *FADS1-2-3* gene cluster [12]. Arachidonic acid, most likely a side chain of PC aa C36:4, is presumably involved in atherosclerotic processes [62,63].

Overlap of associations of a genomic locus with different complex traits can be useful to derive novel hypotheses on possible underlying pleiotropic or causal effects. For instance, recent highly powered meta-analyses have systematically compared the association of type 2 diabetes loci with correlated glycemic (fasting glucose, fasting insulin, 2-h glucose, HbA1C, and others) and metabolic traits (BMI, lipids, and others) [24,25,64-66] in an attempt to better characterize physiologic processes underlying associations at these loci. A similar degree of overlap has been characterized at serum lipid and coronary artery disease loci [16]. While these efforts have provided first important insights into pathophysiologic correlates at disease variants, observed correlations at a locus may often reflect shared environmental effects or confounding rather than causal relations between traits. Distinguishing causality from correlation in these contexts is essential to identify modifiable causes of disease and to unearth new avenues for therapeutic intervention.

The advantage of using metabolites as intermediate phenotypes is that they are more proximal to genes and biological pathways than downstream phenotypes or clinical endpoints [11], ensuring more statistical power to detect genetic associations compared to more complex lipid traits. Furthermore, analysis of metabolites provides the opportunity to dissect complex metabolic pathways into their components. We showed here that through appropriate statistical tools and prioritization strategies we can begin to dissect causal relationships. Although our inferences are limited by the lipid-focused content of the Biocrates metabolomic panel and by the study power, it is foreseeable that information relevant to this and other physiological context can be obtained by applying similar approaches to broader metabolite panels and larger study sizes.

Importantly, we demonstrate that our results are robust in two independent populations and recapitulate a known biological process. For instance, the most plausible path model at *FADS1* predicts that phosphatidylcholines mediate the association between SNP rs174546 and TG. *FADS1* encodes a fatty acid desaturase regulating the desaturation of fatty acids by the addition of a fourth double bond between carbons of the fatty acyl chain [59-61], a role compatible with the observation in this study. This provides proof-of-principle evidence that these approaches deliver robust and interpretable evidence. We further discriminated path models connecting rs1260326 in *GCKR* to TC through phosphatidylcholines. *GCKR* encodes a glucokinase regulatory protein that inhibits glucokinase in liver and pancreatic islet cells by binding non-covalently to form an inactive complex with the enzyme. The locus has been shown to have a pleiotropic effect on multiple cardio-metabolic phenotypes [15,24,52-56]. We postulate here that *GCKR* SNP rs1260326 affects TC through regulation of phosphatidylcholine metabolism, a hypothesis that needs to be validated in experimental settings.

Conditional analysis is a commonly used approach to show dependencies between the variables of the unadjusted model and the variable being adjusted for. However, the different results between unadjusted and adjusted models might be due to reverse causation or confounding rather than causation. One of the most widely applied causal inference approaches is MR. If the direction of the association is previously known between two variables (for example, a metabolite and a lipid in a SNP-MET-LIP set), MR can measure the extent of the unconfounded causal relationship using genetic variants as instrumental variables. However, in some –omics level studies, the direction of the association among variables cannot be easily assumed. To overcome this limitation of MR, we also applied SEM, which evaluates each hypothesis based upon the directional relationship of variables by comparing it with all possible hypotheses and infers the most likely causal relationship. By applying both SEM and MR to our dataset, we obtained significant support for our hypothesis on the direction and the degree of association in each SNP-MET-LIP set. Our framework suggests the usefulness of combined statistical methods as an exploratory tool to infer causal relationship from high-dimensional molecular data.

Although our approach helps to infer causation statistically, it has limitations. In MR, the validation for all of the assumptions is not always feasible, although its violation could increase the bias [67]. MR also has relatively low statistical power and may be affected by weak instrument bias as only the small percentage of phenotypic variance is explained by single (or often multiple) genotypes for most complex traits. Using weak genetic instruments may cause biases [68]. Furthermore, traditional MR is limited in only testing specific sets of hypotheses. SEM provides a hypothesis-free approach that is complementary to MR, as it enumerates all possible models and infers causality from the most likely model. However, it may mislead causal inference in the presence of unknown confounders [46] or measurement errors [69]. Finally, the use of BIC scores to select the most likely model may represent a further limitation of the model. A recent study showed that the new causal model selection test (CMST test) outperforms BIC in terms of statistical precision, although it has lower statistical power [20]. More generally, both MR and SEM in our suggestive framework are designed to detect only linear relationships and targeted on a small set of variables, which were statistically significantly cross-associated with one another (that is, SNP-MET-LIP set). Thus, this framework cannot be readily applied to complex dataset where hundreds or thousands of variables are linearly and non-linearly related.

Recent papers based on Gaussian graphical models or Bayesian networks [42,43,70-72] take into account all the observed variables of a dataset to infer direct correlation or directional correlation. For example, the IDA method (*Intervention-calculus when the DAG is Absent*) estimates total causal effects from all the observed variables using PC algorithm and intervention calculus [42]. Although these approaches are still at risk of being misled by unknown confounders and measurement errors, in contrast to MR, adding more meaningful observed variables to the model may help to robustly handle unaccounted-for factors or high correlations among variables. Our future studies will include improving the statistical framework shown here, to be more adequate for increasingly multiple high-dimensional datasets (such as -omics datasets). On another note, well-designed simulation studies would be beneficial to understand and hopefully overcome the limitations of each of causal methods introduced in this paper.

## Conclusions

Biological systems are clearly far more complex than relatively simple sets of equations. However, new insights on underlying biological processes can be obtained from the analysis of data generated in a highly standardized manner and the careful choice of model variables. We showed that, with the use of appropriate statistical instruments, we could dissect the contribution of metabolites assessed through high-throughput molecular profiling to complex biological pathways. The application of these methods to loci identified in large-scale associations of genome-wide SNP data will provide powerful tools for dissecting metabolic pathways at a wide range of complex trait loci. Preliminary studies exploring metabolic signatures associated with hypertension [73,74], myocardial ischemia [75], and others [76,77] will aid the dissection of genetic and environmental causes of cardio-metabolic disease. The application of metabolomics profiling to samples from large population cohorts, stratified by known risk factors or exposures, may thus provide alternative and powerful designs to test causal relationships while minimizing the impact of clinical confounding variables [77], and new avenues to improve prediction of clinical outcomes.

## Competing interests

The authors declare that they have no competing interests.

## Authors’ contributions

Data analysis: SYS, AKP, CG, and NS. Manuscript preparation: SYS, AKP, CG, and NS. Provision of data or materials: SW, GZ, WRM, KSS, AD, AP, EG, CP, RWS, H-EW, MHdeA, TI, JA, PD, TDS, and KS. All authors read and approved the final manuscript.

## Supplementary Material

Additional file 1: Table S1 Description of study samples. **Table S2.** Characteristics of metabolites analyzed in this study. **Table S3.** SNP quality metrics in KORA and TwinsUK. **Table S4.** Metabolite-lipid correlation metrics. **Table S5.** Results of conditional analysis. **Table S6.** Results of Mendelian randomization. **Table S7.** Results of structural equation modeling. **Table S8.** Skewness of metabolites in 38 significant SNP-MET-LIP sets tested. Click here for file

Additional file 2: Figure S1 Metabolite-lipid correlation heat maps. Heat map plot of metabolite-lipid correlation combined with a hierarchical clustering to show six main groups of metabolites showing similar patterns of correlation with main lipids. The groups are separated by the heavy black line in the heat map and labeled 1 to 6 from top to bottom. The metabolites in each group can be found in the table below. Click here for file
