# Supplementary material for: Interrogating causal pathways linking genetic variants, small molecule metabolites, and circulating lipids
Source: Genome Med. 2014 Mar 28;6(3):25. doi: 10.1186/gm542 (PMC4062056; doi:10.1186/gm542)
Supplement: Additional file 2: Figure S1 — Metabolite-lipid correlation heat maps. Heat map plot of metabolite-lipid correlation combined with a hierarchical clustering to show six main groups of metabolites showing similar patterns of correlation with main lipids. The groups are separated by the heavy black line in the heat map and labeled 1 to 6 from top to bottom. The metabolites in each group can be found in the table below. [file gm542-S2.pptx]

## Slide 1
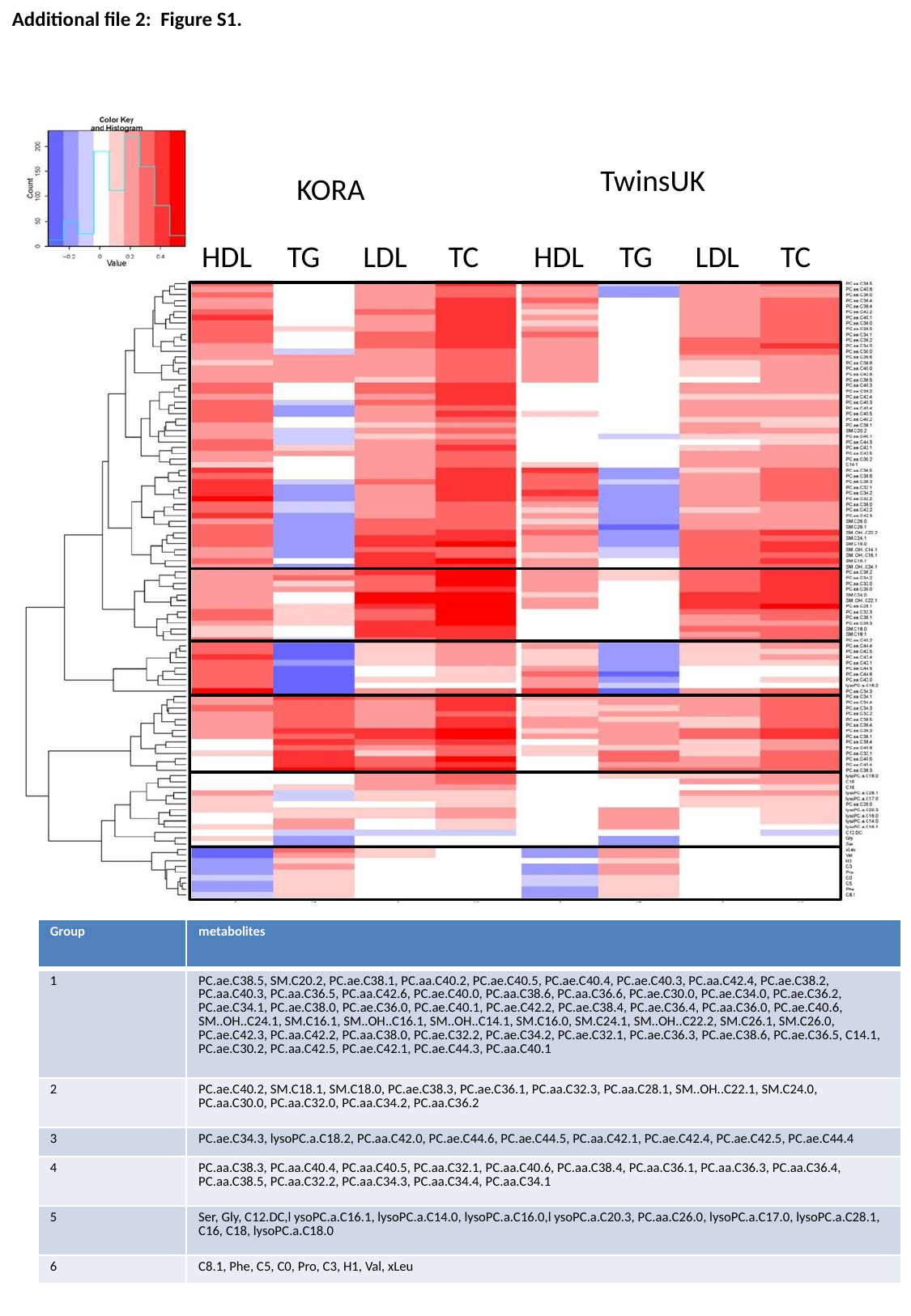

Additional file 2: Figure S1.
TwinsUK
KORA
HDL
TG
LDL
TC
HDL
TG
LDL
TC
| Group | metabolites |
| --- | --- |
| 1 | PC.ae.C38.5, SM.C20.2, PC.ae.C38.1, PC.aa.C40.2, PC.ae.C40.5, PC.ae.C40.4, PC.ae.C40.3, PC.aa.C42.4, PC.ae.C38.2, PC.aa.C40.3, PC.aa.C36.5, PC.aa.C42.6, PC.ae.C40.0, PC.aa.C38.6, PC.aa.C36.6, PC.ae.C30.0, PC.ae.C34.0, PC.ae.C36.2, PC.ae.C34.1, PC.ae.C38.0, PC.ae.C36.0, PC.ae.C40.1, PC.ae.C42.2, PC.ae.C38.4, PC.ae.C36.4, PC.aa.C36.0, PC.ae.C40.6, SM..OH..C24.1, SM.C16.1, SM..OH..C16.1, SM..OH..C14.1, SM.C16.0, SM.C24.1, SM..OH..C22.2, SM.C26.1, SM.C26.0, PC.ae.C42.3, PC.aa.C42.2, PC.aa.C38.0, PC.ae.C32.2, PC.ae.C34.2, PC.ae.C32.1, PC.ae.C36.3, PC.ae.C38.6, PC.ae.C36.5, C14.1, PC.ae.C30.2, PC.aa.C42.5, PC.ae.C42.1, PC.ae.C44.3, PC.aa.C40.1 |
| 2 | PC.ae.C40.2, SM.C18.1, SM.C18.0, PC.ae.C38.3, PC.ae.C36.1, PC.aa.C32.3, PC.aa.C28.1, SM..OH..C22.1, SM.C24.0, PC.aa.C30.0, PC.aa.C32.0, PC.aa.C34.2, PC.aa.C36.2 |
| 3 | PC.ae.C34.3, lysoPC.a.C18.2, PC.aa.C42.0, PC.ae.C44.6, PC.ae.C44.5, PC.aa.C42.1, PC.ae.C42.4, PC.ae.C42.5, PC.ae.C44.4 |
| 4 | PC.aa.C38.3, PC.aa.C40.4, PC.aa.C40.5, PC.aa.C32.1, PC.aa.C40.6, PC.aa.C38.4, PC.aa.C36.1, PC.aa.C36.3, PC.aa.C36.4, PC.aa.C38.5, PC.aa.C32.2, PC.aa.C34.3, PC.aa.C34.4, PC.aa.C34.1 |
| 5 | Ser, Gly, C12.DC,l ysoPC.a.C16.1, lysoPC.a.C14.0, lysoPC.a.C16.0,l ysoPC.a.C20.3, PC.aa.C26.0, lysoPC.a.C17.0, lysoPC.a.C28.1, C16, C18, lysoPC.a.C18.0 |
| 6 | C8.1, Phe, C5, C0, Pro, C3, H1, Val, xLeu |
